# Variations in diagnostic testing utilization in Italy: Secondary analysis of a national survey

**DOI:** 10.1371/journal.pone.0196673

**Published:** 2018-06-12

**Authors:** Pamela Barbadoro, Antonella D’Alleva, Sara Galmozzi, Gemma Zocco, Francesco Di Stanislao, Emilia Prospero, Marcello Mario D’Errico

**Affiliations:** Sezione di Igiene, Medicina Preventiva e Sanità Pubblica - Dipartimento di Scienze Biomediche e Sanità Pubblica - Facoltà di Medicina e Chirurgia – Università Politecnica delle Marche, Ancona, Italy; National Institute of Health, ITALY

## Abstract

**Background:**

According to the principle of horizontal equity, individuals with similar need may have the same possibility of access to health services. The aim of this study is to identify patterns of diagnostic services utilization, in people with, and without chronic disease in Italy.

**Methods:**

Secondary analysis of data from the national survey on Health and use of health care in Italy, carried out in 2013, including 99,497 participants. Multilevel analysis has been used to study the variables associated to diagnostic services utilization.

**Results:**

13.78% of participants have had one diagnostic testing in the four weeks before the interview. In healthy people, utilization of diagnostic testing is reduced in people with low educational level (OR 0.75; 95%CI 0.67–0.84), in housewives (OR 0.66; 95%CI 0.51–0.87), or in those unable to work (OR 0.48; 95%CI 0.26–0.87), while increased in those perceiving a worse health status (up to OR 4.00, 95%CI 2.00–8.01 in very bad health). In people afflicted with chronic disease, access to diagnostic assessment is impaired by educational level (OR 0.69; 95%CI 0.61–0.78) and low household income (OR 0.75; 95%CI 0.58–0.97), while it is increased in the presence of a ticket exemption (OR 1.55, 95%CI 1.42–1.68), and fixed-term occupation (OR2.28, 95%CI 1.31–3.95). Being former-smokers in associated to an increased utilization of services in both groups.

**Conclusions:**

Despite a universal and theoretically egalitarian, public, health care system, variations in diagnostic services utilization are still registered in Italy, both in healthy people and those afflicted by chronic diseases, on socio-economic/occupational basis, and self-perceived health status. Moreover, this significant effect of occupation on healthcare utilization, suggests the need for a comprehensive evaluation of economics in occupational health.

## Introduction

According to the principle of horizontal equity, individuals with similar need may have the same possibility of access to health services [1,2], and the possibility of equity in access to health care is a high priority and integral to the evaluation of health-care system quality [3]. Socioeconomic differences in health are well documented across the European countries [4–6]. In this context, equity in healthcare utilization has increasingly been recognized as an important intermediate step to achieve the final objective of equity in health [7,8]. As stated in the Italian Constitution, the achievement of equitable access to healthcare is a core objective of the National Health care system; however, regional variation in self-perceived health, as well as in other health conditions has been previously registered [9], as well as a lack of horizontal equity in the general population [10]. Moreover, previous studies across different countries evidenced that the access in healthcare may vary even under universal coverage schemes [11,12]. Inequality in the access to health services and in health outcomes have been attributed to ethnicity [13], sex [14,15] and socioeconomic status [16]. However, the presence of chronic disease remains the most important reason to use the healthcare system [17]; thus, the aim of this study is to identify inequalities in diagnostic services utilization, and the role of Regional policies, in people with, and without, chronic diseases in a national representative sample of people living in Italy.

## Materials and methods

### Study design and sample

Data were drawn from the survey “Health and use of health care in Italy,” a national cross-sectional survey conducted every five years by the Italian National Institute of Statistics (ISTAT). The health survey is performed to monitor health care needs and use of healthcare services, and collects information about perceived health status, disease symptoms, chronic disability conditions, and social determinants of health. The data used in this analysis is owned by ISTAT and the authors do not have permission to make it publicly available; however, interested researchers would be able to access these data by motivated request for permission addressed to the ISTAT President by means of the Contact Centre (at http://contact.istat.it/).

The last edition of the survey, carried out between September 2012 and June 2013, gathered data on 49,811 families and 119,037 individuals [18]. A stratified multi-stage probability design was used to select a sample using municipal lists of households. In the first stage, municipalities were the primary sampling units. Municipalities were selected from 67 strata defined on a regional basis and based on population size. The second stage of the sample design involved clustering households from municipality lists. The sampling unit was a household of persons living together with legal, affective, or family relationships, without regard to number of persons in the household. Within each municipality, a minimum of 30 households were randomly selected to be included in the study. Sampling continued without replacement until the required sample size was achieved. Exclusion criteria were: died family members, residence outside of Italy or in a residential care facility, address of residence not available. Each participant in the survey first completed a self-administered questionnaire, and then had a face-to-face interview with ISTAT data collectors. Every participant provided written informed consent.

The present study focused on 99,479 subjects of 18 years of age or more at the time of the survey.

### Study variables

The following question was used as the dependent variable for this study: “In the last 4 weeks, did you undergo any diagnostic testing (excluded those that you have done during a hospital admission)?”. The question refers to various kind of diagnostic testing: blood and urinary test, ultrasound, X-ray, computerized tomography (CT), magnetic resonance, mammography, and pap-smear. The analysis had been conducted comparing two groups of population: those who have answered “yes” at the question “did you suffer of chronic disease or long-term health problems?”, thus classified as “affected by chronic diseases”, and participants not affected by any chronic disease.

Socio-economic, and demographic characteristics of the sample, including sex, age, marital status, employment features, level of education, and citizenship have been analysed. In particular, among the socio-economic variables, the following characteristics have been considered: sex (male; female); age group (18–24 years; 25–44 years; 45–64 years; 65–74 years; more or equal than 75 years); area of residence (Northwest; Northeast; Center; South; the Islands); citizenship (Italian; foreign); marital status (single; married; separated or divorced; widowed); family unit (person living alone; couple with children; couple without children; single-parent family); educational level (high, for people having university degree; medium–high, for people with secondary degree; low, for people having intermediate school degree; no title, for people with elementary degree or without instruction); employment status (employed; between jobs; searching for the first job; housewife; student; unable to work; retired; other employment status); kind of job (employee; fixed term contract; self-employed; not working); working position (director; junior manager; office worker; workman; apprentice; at home worker; not working, or not dependent work); self-perceived household income (good, adequate, insufficient, completely insufficient).

Moreover, clinical variables were considered as follows: presence of disease in the last 4 weeks (no; yes); Medical examination in the last 4 weeks (no; yes); Ordinary admission in the last 3 month (no; yes); Day-hospital / day-surgery in the last 3 months (no; yes); Pap smear in the last 4 weeks (no; yes); Mammography in the last 4 weeks (no; yes); Ticket exempt (no exemption; total exemption; partial exemption); presence of neurosensorial disease, such as blindness, deafness or deafness-muteness (no; yes); Motor disability (no; yes); self-perceived health status (very good; good; intermediate; poor; very poor); Smoking habit (yes; ex-smoker; never smoked); Body Mass Index (normal weight, BMI≥18.5 and <25; underweight, BMI<18.5; overweight, BMI≥25 and <30; and obese, BMI≥30).

### Data analysis

All the factors reported below had been evaluated for their relation to the utilization of diagnostic tests.

Bivariate analyses were performed to analyze the distribution of variables in the sample using chi-square tests, as appropriate. Multilevel logistic regression models were developed to adjust for confounding, and to evaluate which factors were independently associated with the use of diagnostic tests in people with, or without chronic disease (1 when diagnostic tests were performed; 0 when this not occur). The significance level for variables to enter the multilevel logistic regression model was set at ≤0.2, and for removing them from the model at ≤0.4. Analyses were performed with STATA, version 9. The level of significance was set at 0.05. Ethics committee approval for this study was not required, given it uses data made available to researchers by the ISTAT, that collects and manages information in full compliance with the standard regulations.

## Results

The final sample is composed by 99,479 people, afflicted with chronic disease in 29.67% (n = 29,515) of cases; 13.78% (*n* = 13,705) of participants declared having had at least one diagnostic testing in the four weeks before the interview. In particular, 9.43% (n = 6,596) of those not afflicted with chronic disease had undergone a diagnostic testing; whereas 24.09% (n = 7,109) of those afflicted with chronic disease had received a diagnostic test. 83.38% (n = 11,427) of people that had undergone testing, had had a blood test, 46.83% (n = 6,418) a urine analysis, and 49.27% (n = 6,752) a more specialized examination (i.e.: ultrasound, X-ray, computerized tomography, magnetic resonance, mammography, or pap-smear). A distribution of utilization by socio-economic variables is listed in Table 1. Multilevel logistic regression analysis (see Table 2), in people not afflicted by chronic disease, revealed that variables significantly associated with the utilization of diagnostic confirmation were: female gender (OR 1.15; 95%CI 1.08–1.22), marital status as married (OR 1.16; 95%CI 1.04–1.28) compared to single, family unit with a single parent (OR 1.13; 95%CI 1.01–1.27) compared to isolated people. In the same group, socio-economic factors negatively associated with the access to diagnostic testing were: low educational level (ORs ranging from 0.86; 95%CI 0.79–0.94 for subjects with secondary degree, to OR 0.75; 95%CI 0.67–0.84 for subjects with elementary degree, or without education), residence in Southern areas (OR 0.79; 95%CI 0.65–0.96) compared to those living in the North West of Italy, being between jobs (OR 0.70; 95%IC 0.53–0.91), or looking for the first job (OR 0.52; 95%CI 0.37–0.73), being an housewife (OR 0.66; 95%CI 0.51–0.87), or being unable to work (OR 0.48; 95%CI 0.26–0.87) compared to those employed; among the different kind of occupation, being a self-employed was associated to a reduced health care utilization (OR 0.71; 95%CI 0.55–0.93), and, among the employee, being a workman was associated to a reduced utilization (OR 0.69; 95%CI 0.53–0.90) with respect to managerial jobs. Selected clinical variables positively associated with the utilization of diagnostic assessment were: having been ill during the last 4 weeks (OR 1.34; 95%CI 1.26–1.42), having been admitted to the hospital in the last 3 months (ordinary admission OR 2.24; 95%CI 1.94–2.57; day-hospital/day-surgery OR 1.59; 95%CI 1.33–1.90), having a medical examination during the last 4 weeks (OR 4.59; 95%CI 4.33–4.87). Dealing with selected healthy habits, being an ex-smoker was associated to and increased probability of having used diagnostic testing during the previous period (OR 1.20; 95%CI 1.10–1.30) compared to smokers. Analysis has included the study of preventive services utilization, thus having been screened in the last 4 weeks was associated to an increased referral of healthcare utilization (pap smear OR 1.75; 95%CI 1.38–2.21; mammography OR 2.53; 95%CI 2.06–3.10). Among the clinical conditions, being afflicted by a motor disability (OR 1.40; 95%CI 1.13–1.74), and perceiving a poor health status were associated to an increased health care utilization (ORs ranging from 1.19; 95%CI 1.09–1.29 for subject declaring to feel good, to OR 4.00; 95%CI 2.00–8.01 for subject perceiving their health status as very bad) compared to who feels very good. Dealing with the role of partial participation to the public health-care expenditure, having a ticket exemption were associated to an increased health care utilization (complete exemption OR 1.57; 95%CI 1.42–1.72; partial exemption OR 1.52; 95%CI 1.40–1.64).

**Table 1:** Distribution of bivariate associations between demographic, socio-economic, health care services utilization variables, and of diagnostic testing utilization in the sample of 29,515 people afflicted with chronic diseases, and 69,964 people not afflicted with chronic diseases.

	Not afflicted with chronic disease			Afflicted with chronic diseases		
	No.	%	p	No.	%	p
**Sex**			**<0.001**			**0.007**
** 1 Male**	2828	8.13		2922	23.31	
** 2 Female**	3768	10.71		4187	24.66	
**Age class**			**<0.001**			**<0.001**
** 1 18–24 years**	419	5.51		105	15.7	
** 2 25–44 years**	2024	7.85		804	19.96	
** 3 45–64 years**	2422	10.00		2331	22.91	
** 4 65–74 years**	978	13.32		1738	26.90	
** 5 ≥75 years**	753	15.00		2131	26.05	
**Area of residence**			**<0.001**			**0.013**
** 1 Northwest**	1577	10.10		1534	23.68	
** 2 Northeast**	1472	10.73		1466	23.28	
** 3 Central**	1396	11.09		1301	24.84	
** 4 South**	1518	7.71		1850	23.66	
** 5 Islands**	633	7.59		958	26.01	
**Citizenship**			**<0.001**			**0.006**
** 1 Italian**	6273	9.60		6965	24.20	
** 2 Foreign**	323	6.94		144	19.75	
**Marital status**			**<0.001**			**<0.001**
** 1 Single**	1546	6.78		886	19.83	
** 2 Married**	3946	10.57		4268	24.89	
** 3 Separated/Divorced**	536	9.60		489	23.53	
** 4 Widowed**	568	13.41		1466	25.19	
**Family unit**			**<0.001**			**<0.001**
** 0 None unit**	1128	9.77		1917	24.47	
** 1 Couple with children**	3169	8.27		2235	22.16	
** 2 Couple without children**	1686	12.69		2440	26.61	
** 3 Single-parent family**	613	8.98		517	21.32	
**Educational level**			**<0.001**			**0.183**
** 1 University degree**	985	10.62		624	24.33	
** 2 Secondary degree**	2480	8.77		1813	23.71	
** 3 Intermediate school degree**	1784	8.35		1795	23.44	
** 4 Elementary degree or without education**	1347	12.21		2877	24.70	
**Employment status**			**<0.001**			**<0.001**
** 1 Employed**	3019	8.72		1613	21.08	
** 2 Between jobs**	477	8.10		335	21.64	
** 3 Searching the first job**	102	4.24		57	17.81	
** 4 Housewife**	891	9.75		1448	23.98	
** 5 Student**	278	6.05		77	15.88	
** 6 Unable to work**	17	8.02		224	25.00	
** 7 Retired**	1673	14.14		3253	27.01	
** 8 Other employment status**	139	11.00		102	19.10	
**Occupation**			**<0.001**			**<0.001**
** 1 Employee**	2289	9.05		1198	21.21	
** 2 Fixed term contract**	69	9.53		43	29.86	
** 3 Self-employed**	661	7.68		372	20.02	
** 4 Not working**	3577	10.12		5496	25.14	
**Working position of dependent workers**			**<0.001**			**<0.001**
** 1 Director**	84	12.14		35	18.13	
** 2 Manager**	174	11.55		88	22.39	
** 3 Employee**	1126	10.46		604	22.99	
** 4 Workman**	876	7.37		462	19.35	
** 5 Apprentice**	27	6.85		6	17.14	
** 6 At home worker**	2	4.26		3	25.00	
** 7 Not working or not dependent work**	4307	9.64		5911	24.77	
**Self-perceived household income**			**<0.001**			**0.624**
** 1 Good**	136	9.53		117	25.22	
** 2 Adequate**	4177	9.78		3940	24.33	
** 3 Insufficient**	1956	8.99		2548	23.75	
** 4 Completely insufficient**	327	8.00		504	23.65	
**Acute Illness in the last 4 weeks**			**<0.001**			**<0.001**
** 1 No**	4028	7.24		2439	16.84	
** 2 Yes**	2568	17.89		4670	31.07	
**Medical examination in the last 4 weeks**			**<0.001**			**<0.001**
** 1 No**	2579	4.85		1497	11.18	
** 2 Yes**	4017	23.89		5612	34.80	
**Hospital admission in the last 3 months**			**<0.001**			**<0.001**
** 1 No**	6244	9.08		6306	22.79	
** 2 Yes**	352	29.78		803	43.52	
**Day-hospital / day-surgery in the last 3 months**			**<0.001**			**<0.001**
** 1 No**	6405	9.26		6522	23.13	
** 2 Yes**	191	24.77		587	44.60	
**Pap smear in the last 4 weeks**			**<0.001**			**<0.001**
** 0 No**	6486	9.33		7024	23.98	
** 1 Yes**	110	22.87		85	38.81	
**Mammography in the last 4 weeks**			**<0.001**			**<0.001**
** 0 No**	6435	9.27		6973	23.90	
** 1 Yes**	161	27.71		136	39.77	
**Ticket exempt**			**<0.001**			**<0.001**
** 1 No exemption**	4289	7.91		1770	17.40	
** 2 Total exemption**	1198	14.95		2934	27.81	
** 3 Partial exemption**	1109	14.35		2405	27.35	
**Neurosensorial disease**			**<0.001**			**0.001**
** 1 No**	6475	9.35		6546	23.86	
** 2 Yes**	121	16.64		563	27.13	
**Motor disability**			**<0.001**			**<0.001**
** 1 No**	6468	9.34		6017	23.26	
** 2 Yes**	128	18.55		1092	29.98	
**Self-reported Health status**			**<0.001**			**<0.001**
** 1 Very good**	908	5.57		61	12.58	
** 2 Good**	3721	9.22		1136	16.19	
** 3 Intermediate**	1852	14.45		3422	23.82	
** 4 Poor**	97	21.56		1954	32.00	
** 5 Very poor**	18	34.62		536	34.83	
**Smoking habit**			**<0.001**			**<0.001**
** 1 Smoker**	1262	7.82		1027	21.05	
** 2 Former-smoker**	1775	12.38		2521	26.41	
** 3 Never smoked**	3559	9.01		3561	23.60	
**Body mass index**			**<0.001**			**0.007**
** 1 Normal weight**	3431	8.92		2878	23.16	
** 2 Under weight**	214	9.40		212	26.57	
** 3 Overweight**	2216	9.75		2775	24.47	
** 4 Obese**	735	11.33		1244	25.12	

**Table 2:** Multilevel logistic regression model for estimate of independent variables associated with the utilization of diagnostic testing in a sample of 29,515 people afflicted with chronic diseases, and 69,964 people not afflicted with chronic diseases.

	Not afflicted by chronic disease			Afflicted by chronic diseases		
	OR	95% CI	p	OR	95% CI	p
**Sex**						
**1** Male	1			1		
**2** Female	**1.15**	**1.08–1.22**	**<0.001**	1.06	0.99–1.14	0.092
**Age class**						
**1** 18–24 years	1			1		
**2** 25–44 years	1.05	0.90–1.22	0.519	1.00	0.75–1.35	0.990
**3** 45–64 years	1.14	0.97–1.34	0.117	1.04	0.77–1.42	0.790
**4** 65–74 years	0.96	0.79–1.17	0.701	1.01	0.74–1.40	0.931
**5** ≥75 years	0.92	0.75–1.14	0.459	0.88	0.64–1.22	0.451
**Area of residence**						
**1** Northwest	1			1		
**2** Northeast	0.92	0.75–1.12	0.406	0.95	0.85–1.05	0.306
**3** Central	1.05	0.86–1.30	0.623	1.00	0.89–1.12	0.974
**4** South	**0.79**	**0.65–0.96**	**0.017**	0.97	0.87–1.07	0.444
**5** Islands	0.79	0.62–1.02	0.073	1.03	0.91–1.17	0.659
**Citizenship**						
**1** Italian	1			1		
**2** Foreign	0.90	0.79–1.03	0.113	0.87	0.71–1.07	0.177
**Marital status**						
**1** Single	1			1		
**2** Married	**1.16**	**1.05–1.28**	**0.005**	1.06	0.92–1.24	0.420
**3** Separated/Divorced	1.11	0.98–1.25	0.097	1.15	0.99–1.32	0.066
**4** Widowed	1.07	0.93–1.23	0.342	1.10	0.97–1.25	0.138
**Family unit**						
**0** Single	1			1		
**1** Couple with children	1.01	0.89–1.13	0.928	1.10	0.93–1.29	0.276
**2** Couple without children	1.10	0.96–1.25	0.160	1.17	1.00–1.38	0.057
**3** Single-parent family	**1.13**	**1.01–1.27**	**0.033**	0.93	0.82–1.04	0.205
**Educational level**						
**1** University degree	1			1		
**2** Secondary degree	**0.86**	**0.79–0.94**	**0.001**	0.92	0.82–1.03	0.144
**3** Junior high school degree	**0.77**	**0.70–0.85**	**<0.001**	**0.79**	**0.70–0.90**	**<0.001**
**4** Elementary degree or without education	**0.75**	**0.67–0.84**	**<0.001**	**0.69**	**0.61–0.78**	**<0.001**
**Employment status**						
**1** Employed	1			1		
**2** Between jobs	**0.70**	**0.53–0.92**	**0.009**	1.13	0.74–1.72	0.578
**3** Searching for the first job	**0.52**	**0.38–0.73**	**<0.001**	1.03	0.62–1.71	0.906
**4** Housewife	**0.66**	**0.51–0.87**	**0.003**	1.11	0.74–1.67	0.617
**5** Student	0.76	0.56–1.03	0.076	1.03	0.61–1.71	0.923
**6** Unable to work	**0.48**	**0.26–0.87**	**0.015**	1.00	0.65–1.54	0.997
**7** Retired	0.84	0.64–1.09	0.187	1.25	0.83–1.87	0.289
**8** Other employment status	0.93	0.68–1.27	0.655	0.90	0.57–1.42	0.643
**Occupation**						
**1** Employee	1			1		
**2** Fixed term contract	0.88	0.61–1.26	0.479	**2.28**	**1.31–3.95**	**0.004**
**3** Self-employed	**0.72**	**0.55–0.93**	**0.011**	1.22	0.81–1.84	0.346
**4** Not working	-	-	-	-	-	-
**Self-perceived household income**						
**1** Good	1			1		
**2** Adequate	0.99	0.82–1.19	0.882	0.90	0.72–1.13	0.365
**3** Insufficient	0.95	0.78–1.15	0.594	**0.77**	**0.61–0.98**	**0.030**
**4** Completely insufficient	0.94	0.75–1.17	0.569	**0.75**	**0.58–0.97**	**0.027**
**Pap smear in the last 4 weeks**						
**0** No	1			1		
**1** Yes	**1.75**	**1.38–2.21**	**<0.001**	**1.87**	**1.39–2.52**	**<0.001**
**Mammography in the last 4 weeks**						
**0** No	1			1		
**1** Yes	**2.53**	**2.06–3.10**	**<0.001**	**1.84**	**1.45–2.33**	**<0.001**
**Ticket exempt**						
**1** No exemption	1			1		
**2** Total exemption	**1.57**	**1.42–1.72**	**<0.001**	**1.55**	**1.42–1.68**	**<0.001**
**3** Partial exemption	**1.52**	**1.40–1.64**	**<0.001**	**1.54**	**1.43–1.66**	**<0.001**
**Motor disability**						
**1** No	1			1		
**2** Yes	**1.40**	**1.13–1.74**	**0.002**	1.05	0.96–1.14	0.317
**Self-perceived Health status**						
**1** Very good	1			1		
**2** Good	**1.19**	**1.10–1.29**	**<0.001**	1.01	0.76–1.35	0.944
**3** Intermediate	**1.40**	**1.27–1.55**	**<0.001**	**1.36**	**1.03–1.81**	**0.033**
**4** Poor	**1.54**	**1.19–2.00**	**0.001**	**1.65**	**1.24–2.21**	**0.001**
**5** Very poor	**4.00**	**2.00–8.01**	**<0.001**	**1.72**	**1.26–2.34**	**0.001**
**Smoking habit**						
**1** Yes	1			1		
**2** Ex-smoker	**1.20**	**1.11–1.30**	**<0.001**	**1.15**	**1.05–1.26**	**0.002**
**3** Never smoked	1.03	0.96–1.11	0.396	1.08	0.99–1.18	0.106
**Body mass index**						
**1** Normal weight	1			1		
**2** Under weight	1.07	0.92–1.25	0.403	1.11	0.93–1.32	0.237
**3** Overweight	1.01	0.95–1.07	0.801	1.04	0.98–1.11	0.201
**4** Obese	1.08	0.99–1.19	0.089	1.04	0.96–1.13	0.378

*Note*. *Adjusted for the presence of chronic illness in the previous 4 weeks*, *Hospital/Day hospital admission*, *during the last three months*, *and medical examination during the last four weeks before the interview*. OR = odds ratio; CI = confidence interval. In bold are reported the ORs with a statistical significance below 0.05.

At multilevel analysis, in people afflicted by chronic diseases (Table 2), diagnostic tests utilization was inversely associated to low educational status (OR 0.79; 95%CI 0.70–0.90 for subjects with junior high school degree; OR 0.69; 95%CI 0.61–0.78 for subjects with elementary degree or without education compared to subject with university degree) and low household income (OR 0.77; 95%CI 0.61–0.97 for subjects with insufficient household income, OR 0.75; 95%CI 0.58–0.97 for subjects with completely insufficient household income compared to people with a good economic situation), instead it is positively associated with fixed term contract job (OR 2.27; 95%CI 1.31–3.95) compared to employed people. Clinical variables positively associated with the utilization of diagnostic testing included having been ill during the last 4 weeks (OR 1.42; 95%CI 1.34–1.51), having been admitted to hospital in the last 3 months (ordinary admission OR 1.72; 95%CI 1.55–1.91; day-hospital / day-surgery OR 1.81; 95%CI 1.61–2.05), having done medical examination in the last 4 weeks (OR 3.34; 95%CI 3.12–3.57). The study of preventive services utilization has highlighted that having been screened in the last 4 weeks is associated, as expected, to the referral of diagnostic test utilization (pap smear OR 1.87; 95%CI 1.39–2.52; mammography OR 1.84; 95%CI 1.45–2.33). Dealing with selected healthy habits, being an ex-smoker was associated to an increased probability of having used diagnostic testing during the previous period (OR 1.15; 95%CI 1.05–1.26) compared to smokers. Poor self-perceived health status was associated to a greater utilization of diagnostic services (ORs ranging from 1.36; 95%CI 1.03–1.81 for subject declaring to perceive a fair health status, to OR 1.72; 95%CI 1.26–2.34 for subject feeling very bad, compared to who feels very well). Dealing with the role of partial participation to the public health-care expenditure, having a ticket exemption (complete exemption OR 1.54; 95%CI 1.42–1.68; partial exemption OR 1.54; 95%CI 1.43–1.66) was associated to and increased healthcare services utilization. No significant differences were found for participants of non-Italian origin in both groups, of healthy and unhealthy subjects.

The multilevel logistic regression model shows a role of residence region in the utilization of diagnostic assessment in people not afflicted by chronic disease (ICC 0.04; 95%CI 0.09–0.20; p<0.001), instead that association was not significant in people afflicted by chronic disease.

## Discussion

In the present study, 13.78% of the sample declared having had at least one diagnostic testing in the four weeks before the interview. Similar results have been registered in Japan, where 21.1% of participants were classified as high-frequency utilizers, with more than six controls over the previous six years, and frequency of testing was related to medical consultations, possibly linking the presence of chronic conditions and utilisation [19]. As expected, an increased utilisation of diagnostic tests occurs in the presence of a clinical need.

Results have shown that the use of diagnostic assessment in people not afflicted by chronic disease is higher in females, even when the analysis has been adjusted for the possible influence of age, and for the participation to regional screening campaigns including pap smear, or mammography, according to previous shreds of evidence [20,21].

It is interesting to observe that single parents are more likely to undergo diagnostic testing compared to people living alone; however, this finding is in contrast with patterns of disruption in healthcare utilisation/access already registered in single parents, found mostly as barriers in different countries [22,23].

People without a job uses diagnostic testing less than current workers; this result is significant in times of occupational crisis in Italy and confirms previous work highlighting unemployment as one of the most potent health inequalities determinants in Italy [24].

It is also interesting to note that self-employed are less prone to use diagnostic assessment compared to the employee, probably for the inclination of independent workers to reduce the absence at work and highlight the role of time management/constraint in healthcare utilisation [25].

Among those who are employed, an important role is played by job qualification. Workers undergo fewer diagnostic tests than directors; this is in accordance with the existence of a socio-economic gradient in favour of those occupied in managing position at work [26–28].

Following previous studies, utilisation of diagnostic test decreases with educational level, thus underlining an essential area of need [13,15,29–31]. This finding reveals that access to diagnostic assessment deviates from the principle of horizontal equity, despite universal and egalitarian public health care system typical of Italy, meaning that patients afflicted by chronic diseases may be not equally supported by the health care system [10].

Participants with the right to a ticket exemption are more likely to receive diagnostic assessment; ticket exemption, in Italy, is justified by some conditions such as low income or being afflicted by specific diseases. The finding of increased access to diagnostic tests in apparently poorer healthy people maybe is particularly important. In fact, a few studies have examined the association between lower income and higher health care utilisation, and these have shown relationships between low income and higher health care expenditures [31], including more out-patient visits [32].

Other variables associated with a higher frequency of use are those relating to personal habits; in fact, former-smokers are more likely to undergo diagnostic assessment compared to smokers, maybe confirming a possible increased attention to health in those who consciously decided to quit an unhealthy lifestyle. Moreover, in present results, BMI is not related to an increased use of diagnostic testing, showing that obesity is not considered as an important risk factor for the development of multiple complications. This finding is consistent with a lack of attention of obese people on health and prevention, a trend that has been already highlighted in the previous study, dealing with influenza vaccination [33].

The present analysis has also highlighted that people with chronic disease and fixed term contract are more likely to undergo diagnostic assessment compared to employees. This could be the result of an emerging phenomenon of frequent layoffs registered in workers afflicted by chronic diseases, often hired with a fixed-term contract. Moreover, this significant effect of occupation on health expenditure, and healthcare utilization, suggests the need for a comprehensive evaluation of economics in occupational health.

Differently to other recent analyses reaffirming the barrier of race/ethnicity in health and healthcare utilization [34,35], a disadvantage in the access of diagnostic services linked to foreign citizenship has not been encountered in the present study. On the other hand, it must be considered that foreigners participating to the questionnaire may represent an élite of those currently present in Italy; in fact, the ISTAT survey fails to reach illegal immigrants and foreigners without a residency permit. One of the limitations of this study may include the lack of information about the waiting time before the testing; future studies might include the analysis of a prolonged waiting list on patients’ access to healthcare services.

## Conclusions

This study shows that in Italy there are still efforts to be run to ensure equity in the use of diagnostic services for all citizens. Therefore, an egalitarian health system should aim at improving protection of these subject, through enhancing prevention activities and practitioner ability to understand unexpressed needs. On the other hand, conscious and appropriate use of the healthcare system should be warranted.
